# For a Model of Self-Citation Governance in *Arquivos Brasileiros de Cardiologia*

**DOI:** 10.5935/abc.20180160

**Published:** 2018-09

**Authors:** Marcos Antonio Almeida-Santos, Deyse Mirelle Souza Santos, Beatriz Santana Prado, José Augusto Barreto-Filho

**Affiliations:** 1 Programa de Pós-graduação em Saúde e Ambiente da Universidade Tiradentes, Aracaju, SE - Brazil; 2 Núcleo de Pós-Graduação em Ciências da Saúde da Universidade Federal de Sergipe, Aracaju, SE - Brazil

**Keywords:** Periodical Index, Periodicals as Topic, Cardiology, Citation Databases, Journal Impact Factor

*Arquivos Brasileiros de Cardiologia* (ABC) has been the official scientific publication of Brazilian Society of Cardiology (*Sociedade Brasileira de Cardiologia, SBC*) since 1948. Since its beginning, ABC has continuously published articles on a wide range of topics in cardiology, becoming the main organ of dissemination of scientific work in Brazil and Portuguese-speaking countries. Since 1950, the articles have been indexed in the main international databases (Institute of Scientific Information - ISI Web of Science; Cumulated Index Medicus - MEDLINE; Pubmed Central; EMBASE; SCOPUS; SCIELO and LILACS),^1^ and are currently published in two languages (English and Portuguese). Thomson Reuters publishing company, owner of the Journal Citation Reports (JCR) statistical database, created the concept of impact factor in 1955, aiming to provide an instrument to evaluate the performance of scientific publications in a comparative and quantitative manner. Impact factor is calculated by dividing the number of citations of articles published in an academic or technical journal indexed in the ISI by the total number of articles published in that journal during the two preceding years. These estimates include approximately 3,300 journals, 200 subject areas and 100 countries.^2^

Impact factor is a metric for journal assessment, widely used by researchers to choose the best journal to submit their papers. However, its validity as an indicator of scientific impact has been questioned due to, among others, self-promoting journal self-citation in attempt to increase their impact factor.^3^ Overuse of self-citation practices is punishable, and caused the exclusion of 5 Brazilian journals from the JCR for one year (2013).^4^ For this reason, efficient control measures of this practice should be developed and implemented by scientific journals that seek to adhere to ethical precepts recommended by the scientific community.

In light of this, we developed an original model of regulation of self-citations of scientific publications, by analysis of ABC publications in terms of internal bibliographic referencing, known as self-citation, considered valid for the impact factor calculation, between 2000 and 2016.

The search was performed on the ABC database available at (http://www.arquivosonline.com.br). Inclusion criteria were the period from 2000 to 2016, and the texts classified as "original articles". Aiming to avoid heterogeneity of data and eventual publication bias, manuscripts classified as "editorials", "letters to the editor", etc. were excluded from the search. Original articles were classified by month, year, total number of references, number of references of articles published in ABC, and number of "valid" references for impact factor calculation, i.e., articles published within two years prior to current publication.

Temporal trend analysis was performed for the number of "valid" publications and its relation to the number of citations in the same journal and the total number of references. Assumption of temporal stability was assessed by rolling window regression, including the following parameters - monthly periodicity, non-recursive sampling (i.e., a fixed window size), overlapping window of subsamples in a six-month range beyond sample size, fixed at 1,875, of "original articles" published between January 2000 and December 2016 in ABC. Rolling window regression was used for analysis of countable data (Poisson distribution), with the number of references considered "valid" for calculation of the impact factor, per month, used as dependent variable, and total number of references per month used as independent variable. Coefficients obtained from successive intervals of six months were exponentiated to represent the "effect size" of temporal series as incidence rate ratio (IRR). Values near 1 (margin of error of 5%) for a pre-established period indicated absence of the effect. Values lower than 1 indicated reduction and values greater than 1 suggested increment in incidence rate, with valid references.

To evaluate potential influence of journal volume proximity, previous trends, future predictions, preference for certain periods of the year and stationary processes, autoregressive integrated moving average (ARIMA) model was selected according to pre-estimation and post-estimation.

Pre-estimation was assessed by periodgrams, correlograms, partial and total autocorrelation plots with 95% confidence interval based on the Q test (or "portmanteau") and the Bartlett's test. Post-estimation was assessed using the Akaike information criterion (AIC) and smoothers for detection of the Gaussian white noise in time series graphs. These are characterized by a trend for asymmetry, lack of correlation with time, and presence of stationary processes. Result of ARIMA model was described according to time operators, such as "lag", "lead" and "difference". used in the analysis of the best performance.

Stability of all parameters selected in ARIMA model was assessed by estimation of eigenvalues and their graphical display inside the unit circle of the inverse root of the ARIMA polynomials. Significance level was set at two-tailed p < 0.05. Statistical analysis was performed using Stata software (version 14.2). Results of the analyses are described in four stages, as follows:

Stage 1. A total of 1,875 articles were analyzed, corresponding to all "original articles" published between 2000 and 2016 in ABC. Table 1 describes the number of references per article, the number of references of articles previously published in ABC and the number of "valid" references for the impact factor calculation. Data were described per year, as mean and standard deviation.

**Table 1:** Total of references per year, number of references of articles published in Arquivos Brasileiros de Cardiologia (ABC) and number of valid references for estimation of the impact factor from 2000 to 2016

Year	References per article	SD	References or articles published in ABC	SD	References of impact	SD
2000	28.11	2.01	1.29	0.28	0.00	0.00
2001	28.06	1.85	1.21	0.21	0.06	0.02
2002	29.44	1.52	1.18	0.22	0.18	0.07
2003	29.29	1.52	1.18	0.22	0.18	0.07
2004	29.14	1.25	1.14	0.18	0.19	0.05
2005	31.32	1.52	1.36	0.20	0.06	0.02
2006	30.25	1.01	1.38	0.16	0.20	0.42
2007	27.88	0.70	1.54	0.17	0.30	0.04
2008	25.85	0.81	1.30	0.17	0.35	0.07
2009	27.27	0.72	1.92	0.20	0.39	0.07
2010	29.07	0.55	2.00	0.16	0.31	0.04
2011	29.24	0.89	1.47	0.18	0.46	0.07
2012	29.19	0.75	2.00	0.20	0.50	0.06
2013	28.41	0.75	1.92	0.19	0.30	0.05
2014	29.70	0.84	2.11	0.24	0.31	0.07
2015	29.23	0.84	1.59	0.20	0.38	0.08
2016	29.17	0.84	1.65	0.22	0.46	0.09
Mean	28.87	0.24	1.61	0.04	0.30	0.01

Data expressed as mean and standard deviation (SD)

Stage 2. Numerical and graphical analysis of autocorrelations did not reveal temporal, periodic or seasonal trends. Similarly, results of the Q-test were compatible with white noise in models in which lags were not included (p = 0.49) and in models with inclusion of up to 20 lags (p = 0.27), i.e., there was a random change of signal, with no temporal trends or autoregressive phenomena associated with such variation.

Stage 3. ARIMA regression models were tested. The model that met the adequacy criteria, yielding the lowest AIC values, used, as parameters, a "p" (lags) of 6, a first-order "d" (difference) of 1, and "m" (or cut offs after lags, in "leads") of 3. All coefficients had a p-value greater than 0.05, including the first-order difference and sigma, which test the hypothesis of variance in time series different from zero. The model stability was considered satisfactory in numeric terms, for showing eingenvalues lower than 1 in absolute number, as well as in graphical terms for showing the inverse root of polynomials inside the pre-established circle.

**Figure 1:**
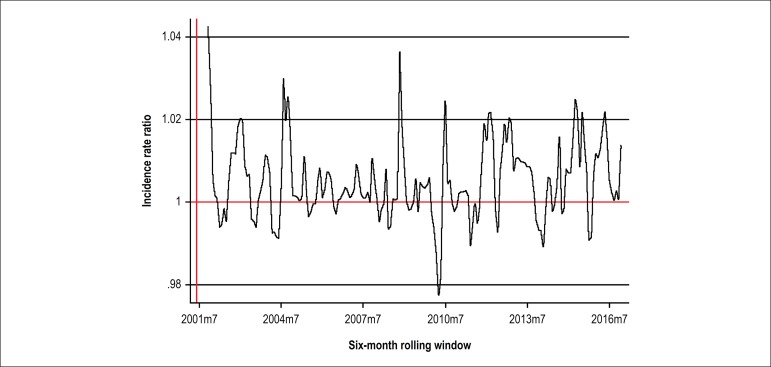
Incidence rate ratio (IRR) of self-citations during the period from 2000 to 2016. Note: results are presented from the year 2001 on, since the year 2000 was used as the starting point of the rolling window regression.

Stage 4. A sequential, six-month window was adopted as parameter in the rolling window regression. A Poisson regression model was used for analysis of countable data, with a robust estimate of both variance and covariance. Distribution of IRR of "valid" references is depicted in Figure 1. An IRR near 1 indicate absence of volatility, i.e., considering the total number of references, the rate of "valid" references did not significantly change throughout the analysis period. Therefore, the pattern of bibliographic referencing remained unchanged in the last 17 years. As shown in graph 1, IRR was extended by nearly 5%, ranging from approximately 0.98 to 1.04 from 2000 to 2016.

Analysis of time series during a 17-year period enabled a detailed description of parameters of bibliographic referencing distribution and self-citation pattern. These data may serve as a basis for future comparisons between different journals and within the same journal. Our main finding was the stationary pattern of self-citations in the bibliographic referencing of original articles, published in ABC between 2000 and 2016, considered "valid" for the impact factor calculation. This suggests that ABC resisted the temptation to encourage self-citation of their reports to increase its impact factor.

In Brazil, the *Coordenação de Aperfeiçoamento de Pessoal de Nível Superior* (CAPES) a Brazilian government agency run by the Ministry of Education, adopts a set of procedures to classify the quality of intellectual production of (professional and academic) Master's degree and doctoral programs, named "Qualis classification". For the same journal, Qualis varies according to the subject area. Qualis classification criteria include the impact factor (or more precisely, the cutoff point) that defines each category,^5^ which is available at Sucupira platform.^6^

ABC is indexed according to formal parameters of assessment of scientific journals, such as its format, the International Standard Serial Number (ISSN), periodicity and main scientific content, presence of a qualified editorial board, peer reviews, and conformity with the norms of the World Association of Medical Editors (WAME), formerly Vancouver group. The journal is also indexed in the National Library of Medicine, Pub Med/Medline, ISI, and SciELO, Lilacs databases, among others.^5,7^

The impact factor of ABC was calculated for the first time by the Journal of Citation Report (Thomson Reuters) in 2010, with a result of 1.315. Since then, values of this bibliometric index, documented by the Web of Science database (ISI) confirmed scientific relevance as well as the scope of the studies conducted in Brazil and of international studies published in ABC. These classifications place ABC at the same level of approximately 30% of international journals in cardiology indexed in the ISI database.^2^ Strategies that may increase the impact factor include the improvement of publication and internationalization criteria of the journal. This has been performed in non-English-speaking countries, by publishing, for example, bilingual editions. Similar strategies have been used in the United States, Mexico and South Korea.^8^

Since its inception in scientific field, Thomson Reuters has developed citation indexes, and compilation of statistical reports, not only in terms of volume of publication but also of the frequency of citations. This used to be done by the Science Citation Index (SCI)^5^ until 1975 and, since then, this process has been continued by Thomson Reuters by means of JCR, as part of the SCI and the Social Sciences Citation Index (SSCI).^10^ JCR provides quantitative tools for classification, assessment, classification and comparison of journals.^9^ The impact factor allows comparisons between different journals year by year.^10^

It's reasonable that the editors of scientific journals strive to improve the scientific quality of the articles to be published, which is achieved, among others, by increasing the number of articles received, by a rigorous selection process and training of reviewers. Not rarely, the prestige and even the survival of a journal depends on the maintenance or, even better, improvement of the impact factor.^11^

Nonetheless, the use of the impact factor has been a matter of controversy in scientific and academic communities. The instrument has been considered inadequate,^12^ of low credibility,^6^ a source of distraction,^13^ a controversial stimulus,^14^ a questionable metrics^15^ to be extinguished^16^ or, at least, a debatable subject.^13^ Also, assessing the scientific quality of the articles in a two-year period may be considered arbitrary.^17^

Despite the criticisms, the impact factor has been used as a bibliometric indicator, i.e., as a discriminating parameter of the relevance of a publication for the scientific community.^12-18^

The use of an easy-to-understand bibliometric indicator represents a valuable contribution, especially considering the increase in the number of electronic journals and online journal access.^10-17]^

However, governance instruments capable of auditing the temporal pattern of self-citation rate and identifying sudden or unexpected increments in the impact factor, possibly associated with inappropriate self-referencing should be developed.

The present model enabled an integrated, dynamic assessment of self-citation rates. During the period from January 2000 to December 2016, there was a stationary pattern of self-citation of original articles published in ABC, which is in accordance with ethical practices in scientific research. Based on our results, we believe that this governance instrument can be of great utility for monitoring the pattern of self-citation practices and increasing transparency of the impact factor as a metric parameter of the quality of scientific journals.
